# Supplementary material for: 3D printing of robotic soft actuators with programmable bioinspired architectures
Source: Nat Commun. 2018 Feb 28;9:878. doi: 10.1038/s41467-018-03216-w (PMC5830454; doi:10.1038/s41467-018-03216-w)
Supplement: Supplementary file 1 — Supplementary Information [file 41467_2018_3216_MOESM1_ESM.pdf]

Supplementary Information:

3D Printing of Robotic Soft Actuators with Programmable Bioinspired Architectures

Manuel Schaffner[1], Lucas Pianegonda[1], Patrick A. Rühs[1], Fergal Coulter[1,2], Jakob Faber[1&], André R. Studart[1&]

[1] Complex Materials, Department of Materials, ETH Zürich, 8093 Zürich, Switzerland

[2] School of Mechanical & Materials Engineering, University College Dublin, Ireland

Supplementary Methods

Analytical Model

For the description of strains and twist angle in dependence of inner pressure, we assume a thin-walled cylinder resulting in a plane stress state in the wall. Similar to the mechanics of fiber composites, we simplify the material architecture of the soft base geometry and the restricting stripes as one homogeneous, orthotropic material. This homogenization leads to a compliance matrix with a preferential stiffness $E_{\parallel}$ in the stripe direction and a smaller $E_{\perp}$ perpendicular to the stripes, which can be written in the material's principal orientation as

$$\begin{pmatrix} \epsilon_1 \\ \epsilon_2 \\ \gamma_{21} \end{pmatrix} = \begin{pmatrix} S_{11} & S_{12} & 0 \\ S_{12} & S_{22} & 0 \\ 0 & 0 & S_{66} \end{pmatrix} \cdot \begin{pmatrix} \sigma_1 \\ \sigma_2 \\ \tau_{21} \end{pmatrix} = \begin{pmatrix} \frac{1}{E_{\parallel}} & -\frac{\nu_{\parallel\perp}}{E_{\perp}} & 0 \\ -\frac{\nu_{\perp\parallel}}{E_{\parallel}} & \frac{1}{E_{\perp}} & 0 \\ 0 & 0 & \frac{1}{G_{\perp\parallel}} \end{pmatrix} \cdot \begin{pmatrix} \sigma_1 \\ \sigma_2 \\ \tau_{21} \end{pmatrix} \quad . \tag{1}$$

The principal direction is typically aligned to the cylinder axis at an angle α, for example $\alpha = 45°$ in the case of the twisting motion mode. In contrast, the stresses in a pressurized cylinder have their principal directions parallel and transverse to the cylinder. They consist of

the axial component $\sigma_x = \frac{p_i D_a}{4 t_a}$ and the tangential component $\sigma_y = \frac{p_i D_a}{2 t_a}$,

with the inner pressure p_i and the average diameter D_a and thickness t_a of the homogenized cylinder. To account for the different directions of these loading components and the material anisotropy, we rotate the latter by polar transformation as commonly known from classical lamination theory:

$$\begin{pmatrix} \epsilon_x \\ \epsilon_y \\ \gamma_{xy} \end{pmatrix} = \begin{pmatrix} \overline{S_{11}} & \overline{S_{12}} & \overline{S_{16}} \\ \overline{S_{12}} & \overline{S_{22}} & \overline{S_{26}} \\ \overline{S_{16}} & \overline{S_{26}} & \overline{S_{66}} \end{pmatrix} \cdot \begin{pmatrix} \sigma_x \\ \sigma_y \\ \tau_{xy} \end{pmatrix} \quad , \tag{2}$$

where each $\bar{S}$ can be derived by polar transformation from the matrix S. Since only normal stresses occur and σ_y is known to be twice as large as σ_x, the strains can be written as

$$\epsilon_x = \overline{S_{11}}\sigma_x + \overline{S_{12}}\sigma_y = \sigma_x(\overline{S_{11}} + 2\overline{S_{12}}) = \frac{p_i D_a}{4 t_a}(\overline{S_{11}} + 2\overline{S_{12}}) \ , \tag{3}$$

$$\epsilon_y = \overline{S_{12}}\sigma_x + \overline{S_{22}}\sigma_y = \sigma_x(\overline{S_{12}} + 2\overline{S_{22}}) = \frac{p_i D_a}{4 t_a}(\overline{S_{12}} + 2\overline{S_{22}}) \ , \tag{4}$$

$$\gamma_{xy} = \overline{S_{16}}\sigma_x + \overline{S_{26}}\sigma_y = \sigma_x(\overline{S_{16}} + 2\overline{S_{26}}) = \frac{p_i D_a}{4 t_a}(\overline{S_{16}} + 2\overline{S_{26}}) \ . \tag{5}$$

The dependence of these rotated compliance matrix components on the fiber angle α can be found readily in literature (*1*) for orthotropic laminate in the direct form:

$$\overline{S_{11}} = \frac{\cos^4(\alpha)}{E_{\parallel}} + \frac{\sin^4(\alpha)}{E_{\perp}} + \frac{1}{4}\left(\frac{1}{G_{\perp\parallel}} - 2\frac{\nu_{\perp\parallel}}{E_{\parallel}}\right)\sin^2(2\alpha) \ ,$$

$$\overline{S_{22}} = \frac{\sin^4(\alpha)}{E_{\parallel}} + \frac{\cos^4(\alpha)}{E_{\perp}} + \frac{1}{4}\left(\frac{1}{G_{\perp\parallel}} - 2\frac{\nu_{\perp\parallel}}{E_{\parallel}}\right)\sin^2(2\alpha) \ , \tag{6}$$

$$\overline{S_{12}} = \frac{1}{4}\left(\frac{1}{E_{\parallel}} + \frac{1}{E_{\perp}} - \frac{1}{G_{\perp\parallel}}\right)\sin^2(2\alpha) - \frac{\nu_{\perp\parallel}}{E_{\parallel}}(\sin^4(\alpha) + \cos^4(\alpha)) \ ,$$

$$\overline{S_{16}} = -\left(\frac{2}{E_\perp} + 2\frac{\nu_{\perp\parallel}}{E_\parallel} - \frac{1}{G_{\perp\parallel}}\right)\sin^3(\alpha)\cos(\alpha) + \left(\frac{2}{E_\parallel} + 2\frac{\nu_{\perp\parallel}}{E_\parallel} - \frac{1}{G_{\perp\parallel}}\right)\cos^3(\alpha)\sin(\alpha) \ ,$$

$$\overline{S_{26}} = -\left(\frac{2}{E_\perp} + 2\frac{\nu_{\perp\parallel}}{E_\parallel} - \frac{1}{G_{\perp\parallel}}\right)\cos^3(\alpha)\sin(\alpha) + \left(\frac{2}{E_\parallel} + 2\frac{\nu_{\perp\parallel}}{E_\parallel} - \frac{1}{G_{\perp\parallel}}\right)\sin^3(\alpha)\cos(\alpha) \ .$$

Up to this point, no assumptions or simplifications were made in addition to plane stress lamination theory. All strain values can be retrieved from either the engineering constants $E_\parallel, E_\perp, G_{\perp\parallel}, \nu_{\perp\parallel}$ for orthotropic materials or the compliance matrix S in the case of more complex materials, such as multilayer arrangements.

To compute displacements instead of strains for our exemplary case, the translation of shear γ_{xy} to twist angle θ is given by:

$$\theta = \frac{2 * \gamma_{xy} * L}{D_a} \ , \tag{7}$$

with L being the cylinder length. The relationship between applied pressure p_i and resulting twist angle θ for a cylinder length L with the stripe angle α can then be expressed as

$$\theta = p_i * \frac{L}{t_a}\left(\left(\frac{2}{E_\parallel} + \frac{2\nu_{\perp\parallel}}{E_\parallel} - \frac{1}{G_{\perp\parallel}}\right)\sin^3(\alpha)\cos(\alpha) + \left(\frac{2}{E_\parallel} - \frac{4}{E_\perp} - \frac{2\nu_{\perp\parallel}}{E_\parallel} + \frac{1}{G_{\perp\parallel}}\right)\cos^3(\alpha)\sin(\alpha)\right) \ . \tag{8}$$

In this relationship, the material parameters to be used as the homogenized properties of the stiff and the soft layers need to be defined. For maximum simplicity, we assume that all parallel stiffness is contributed by the stiff stripes, whereas the soft transverse behavior is given only by the high compliance of the soft layer. By making these assumptions, we can substitute $E_\parallel$ by E^*_{Stiff} and $E_\perp$ by E^*_{Soft}, after normalizing with regards to their volume fraction in the homogenized state:

$$E^*_{\text{Stiff}} = \phi_{\text{Stiff}} E_{\text{Stiff}}$$
$$E^*_{\text{Soft}} = (1 - \phi_{\text{Stiff}}) E_{\text{Soft}} \tag{9}$$

It should be noted that for the transverse modulus E^*_{Soft}, a typical “serial” rule of mixture, as it would be expected for composites, is not suitable here due to the alternating thickness topology. Instead, the normalization of the transverse modulus accounts for the reduced cross-section bearing transverse and shear loads. Additionally, we assume a Poisson’s ratio $\nu = 0.5$ for all elastomeric materials. This strong approximation sacrifices some accuracy especially for the anisotropic Poisson ratio, but greatly simplifies the final relationship that describes the twisting angle:

$$\theta = \frac{p_i L}{t_a}\left(\frac{1}{E^*_{\text{Stiff}}} - \frac{1}{E^*_{\text{Soft}}}\right)(3\sin^3(\alpha)\cos(\alpha) + \cos^3(\alpha)\sin(\alpha)) \ . \tag{10}$$

The geometrical values used for the prediction of the 45° robotic actuator were measured on the printed prototype and are displayed in Supplementary Table 1.

Supplementary Table 1: Geometry and material parameters used for analytical prediction of pressure-twist coupling. Identical values - where applicable - were employed in the finite element simulations.

α	stripe reinforcement angle	45 °
D_a	average diameter	22.6 mm
t_a	average thickness	1.55 mm
L	length	100 mm
ϕ_{Stiff}	volume fraction stiff phase	43.6 %
E^*_{Stiff}	parallel stiffness	1483.6 N/mm²
E^*_{Soft}	transverse stiffness	73.3 N/mm²
θ/p_i	twist per pressure	47.96 °/kPa

Supplementary Note 1

In addition to the actuator shown in Figure 3B, a twisting actuator with a lead angle of 30° was also fabricated and tested (Supplementary Figure 1).

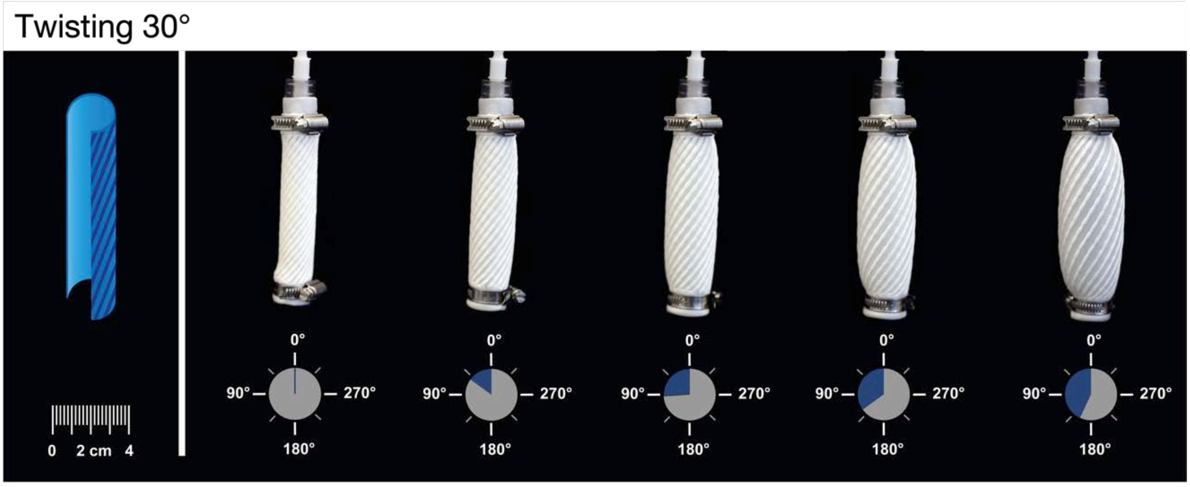

Supplementary Figure 1: Twisting soft actuator with 30° lead angle. The twisting actuator displays a fiber lead angle of 30° with respect to the long axis. The twisting angle increases with the applied internal pressure.

The conformability of the grabber actuator is demonstrated in Supplementary Figure 2, using objects with different cross-section geometries.

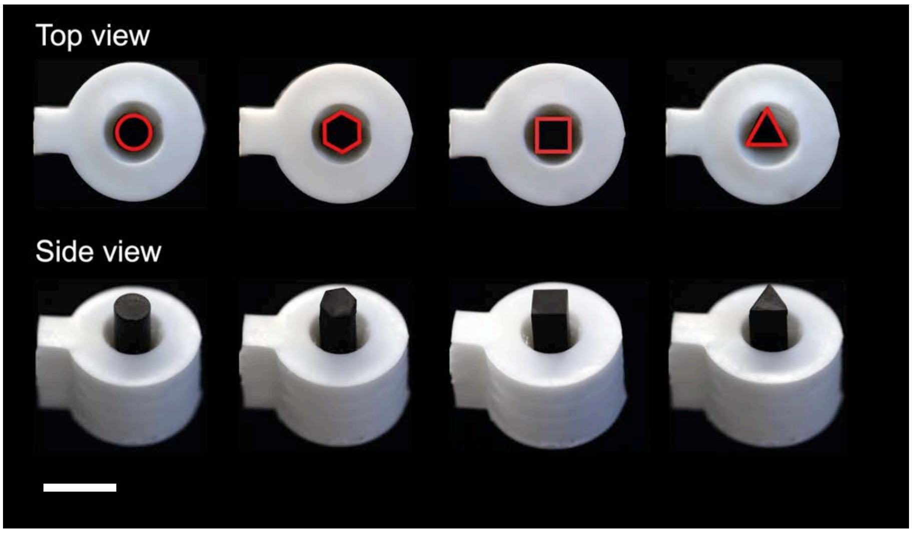

Supplementary Figure 2: Grabbing soft actuator. A grabbing pneumatic actuator holds and seals objects with different shapes, making it a useful tool to handle fragile objects or to control mass flow through the central channel. Round, hexagonal, rectangular and triangular geometries are grabbed and tightly sealed. Scale bar is 2 cm.

A bending actuator was fabricated using stiff stripes printed on top of a soft cylinder (Supplementary Figure 3). While twisting and contraction are programmed by maintaining a fixed lead angle throughout the entire cylinder surface, bending was achieved by combining ϕ values of 0 and 90° in one half of the underlying cylinder.

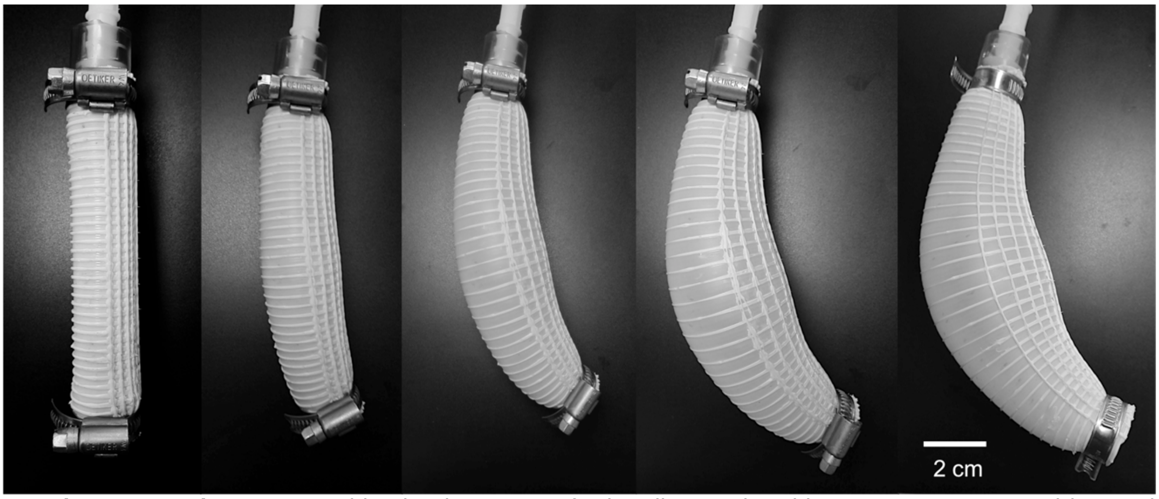

Supplementary Figure 3: Combined stripe pattern for bending mode. This actuator was created by combining longitudinal (α = 0°) and transverse (α = 90°) stiff stripes on one half of the soft silicone cylinder, resulting in complimentary strain and thus bending motion.

Experimental data on the dynamic actuation behavior of the pneumatic contractor (Figure 3A) in shown in Supplementary Figure 4. Using a computer controlled valve, we pressurized the contractor with 0.4 bar at a frequency of 1 Hz. We recorded the contractile displacement at the free end using a laser distance measuring system (Keyence, LK-G5000). The slight deviations in the waveform of

individual cycles are attributed to the dynamics of the pressure control system. The observed actuation speeds were 7.5 mm/s during inflation and 18 mm/s during deflation. In both cases, the air supply and hose diameter were the limiting factors. This places the Silink soft robots among the fastest soft robotic systems (2), as there are no intrinsic limits for the actuation speed such as creep, capillary dynamics, or mass and heat transfer.

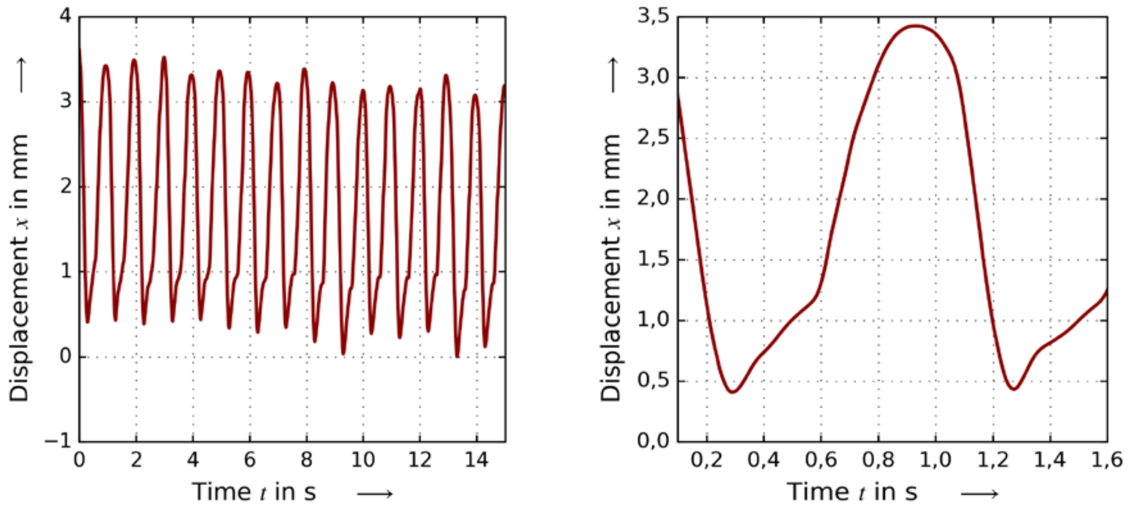

a) b)

Supplementary Figure 4: Dynamic behavior of contracting motion mode. a) Cyclic behavior at actuation using 0.4 bar and 1 Hz. b) Inflation phase at 7.5 mm/s and deflation phase at 18 mm/s.

Supplementary References

1. Jones, R. M. *Mechanics of composite materials*. (Springer, 1975).

2. Mosadegh, B. *et al.* Pneumatic Networks for Soft Robotics that Actuate Rapidly. *Adv. Funct. Mater.* **24,** 2163–2170 (2014).
